# Supplementary material for: West London Healthy Home and Environment (WellHome) Study: Protocol for a Community-Based Study Investigating Exposures Across the Indoor-Outdoor Air Pollution Continuum in Urban Communities
Source: Int J Environ Res Public Health. 2025 Feb 10;22(2):249. doi: 10.3390/ijerph22020249 (PMC11855092; doi:10.3390/ijerph22020249)
Supplement: Supplementary file 1 [file ijerph-22-00249-s001.zip › Supplementary File 4.pdf]

Supplementary File 4

Quantitative profiling of social health inequalities and policy disconnects using toxicological paradigms (WP3)

Methods: Indoor PM samples will be collected from diverse households in West London, capturing a range of exposures based on factors such as area deprivation, seasonality, housing type, and proximity to traffic sources. To capture PM mass sufficient for toxicological analysis, a multi-stage high volume sampler (TSI high flow impactor), collecting PM0.1, PM0.1-2.5 and PM2.5-10 fractions will be used. Sampling will be performed over a 5-day period, only during the daytime, including the periods when cooking is taking place to reduce the inconvenience and burden on the participating households. In addition, source specific PM samples, reflecting indoor PM sources: cooking and cleaning product derived aerosols, plus wood burning and diesel exhaust derived PM will be used, derived from the partner HIP-Tox study [1]. The collected PM samples will undergo enhanced chemical characterisation, including:

(1) Metallomic profiling - total metal and metalloid concentrations, speciation of key redox active metals (e.g., Fe and Cu), and the bioavailable fraction using ICP-MS and chromogenic chelators.
(2) Organic analysis – Qualitative analysis of organic compounds using GC-MS, focusing on compounds with known toxicological relevance.
(3) Bioaerosol characterization – characterisation of the microbiome composition of PM samples using shotgun metagenomic sequencing, and quantitation bacterial endotoxin, lipoteichoic acid, and β-glucan levels.
(4) Oxidative potential assessment - measurement of the intrinsic and cellular oxidative potential of PM samples.
(5) Microplastic analysis: Identify and quantify microplastic particles and fibers using pyrolysis TOF-MS and Raman microscopy.

In vitro toxicological screening: We will expose primary bronchial epithelial cells from healthy and asthmatic donors to PM samples at sub-cytotoxic concentrations. and assess acute and repeated exposure responses, including changes in inflammatory markers (e.g., IL-6, CXCL8, GM-CSF), epigenetic modifications, and cellular oxidative stress.

In vivo toxicological evaluation: We will nebulize PM samples into the noses of asthmatic volunteers and assess upper airway responses, including Damage-Associated Molecular Pattern (DAMP) release (High Mobility Group Box 1 (HMGB1), S100 proteins), cytokine expression (e.g., IL-6, IL-13, IL-17A), and urate concentrations.

Evaluation of PM mixtures indicative of time activity profiles: Using the PM samples outlined above we will design blended PM samples to represent individual participants' time activity

patterns, incorporating indoor and outdoor PM samples in proportions that reflect the time spent in each environment. The composition of these PM samples will then be adjusted to simulate the effects of behaviour change interventions, such as avoiding busy roads, increasing ventilation, or using air purifiers. These blended samples will then be test in the in vitro and in vivo models to determine whether the altered composition reduces the immunotoxicty of the PM mixtures.

Expected outcomes: (1) Identification of key pollutants/pollutant sources contributing to health risks in individuals with asthma across the outdoor-to-indoor continuum. (2) Evaluation of the effectiveness of air pollution mitigation measures. (3) Development of an experimental approach allowing the integration of hazard evaluation, exposure and behaviour change to inform evidence-based policies to improve air quality and public health.

Overall, this in vitro and in vivo hazard evaluation work package will provide valuable insights into the health impacts of air pollution and inform the development of effective interventions to protect vulnerable populations. By combining advanced analytical techniques, in vitro and in vivo models, and rigorous data analysis, this project will contribute to a better understanding of the complex relationship between air pollution and human health.

Reference

1. Faherty, T., et al., *HIPTox—Hazard Identification Platform to Assess the Health Impacts from Indoor and Outdoor Air Pollutant Exposures, through Mechanistic Toxicology: A Single-Centre Double-Blind Human Exposure Trial Protocol*. International Journal of Environmental Research and Public Health, 2024. **21**(3): p. 284.
