# Supplementary material for: West London Healthy Home and Environment (WellHome) Study: Protocol for a Community-Based Study Investigating Exposures Across the Indoor-Outdoor Air Pollution Continuum in Urban Communities
Source: Int J Environ Res Public Health. 2025 Feb 10;22(2):249. doi: 10.3390/ijerph22020249 (PMC11855092; doi:10.3390/ijerph22020249)
Supplement: Supplementary file 1 [file ijerph-22-00249-s001.zip › Supplementary File 1.pdf]

Supplementary File 1

General Questionnaires

- ## General Household Questionnaire 1 (Visit 1)

This questionnaire should take about 30 minutes to complete.

Consent: I have understood the information provided in the Participant Information Sheet and I agree to take part in this survey

○ Yes

○ No

Home ID____

Date____

SECTION A) To be completed by the researcher *(if possible)*

A1. What type of home is this?

- ◯ Detached house
- ◯ Semi-detached house
- ◯ Bungalow
- ◯ Terraced (including end -terrace)
- ◯ A flat, maisonette or apartment- in a purpose -built block of flats or tenement
- ◯ A flat, maisonette or apartment- part of converted or shared house (including bedsits)
- ◯ A flat, maisonette or apartment- part of another converted building (for example, former school, church or warehouse)
- ◯ A flat, maisonette or apartment - in a commercial building (for example, in an office building, hotel or over a shop)
- ◯ Chalet/mobile home

A2. When was this home built?

- ◯ Before 1914
- ◯ 1914 -1944
- ◯ 1945 -1964
- ◯ 1965 - 1990
- ◯ 1991- or later
- ◯ I don't know

A3. Which floor does the participant live in?

____

A4. How far away from this home is the nearest green space area? (e.g. park, playing field, public garden, woodland, or other green space)

- ○ Less than 5-minute walk
- ○ Within a 5–10-minute walk
- ○ Within a 11–20-minute walk
- ○ Within a 21–30-minute walk
- ○ More than 30 min walk

A5. What do you see when you (researcher) look out through the window(s) from the main living area?

- ○ Predominantly grey space, including man-made materials, such as roads and buildings
- ○ Predominantly green space e.g. trees, fields and bushes
- ○ Both grey space and green space
- ○ Didn’t have access to living room

Section B) General Questions About Your Home

B1. Have you lived at your current address for less than 5 years?

- ○ Yes (1)
- ○ No (2)

Display This Question:

If Section B) General Questions About Your Home B1. Have you lived at your current address for less... = Yes

B2. What was your previous postcode?

____

Display This Question:

If Section B) General Questions About Your Home B1. Have you lived at your current address for less... = No

B3. How long have you lived at your current address?

____

B4. Does your household own or rent this accommodation?

- ○ I/we own it or live with the person who owns it (includes homes bought with a mortgage)
- ○ It is rented from the local council
- ○ It is rented from a housing association or housing trust
- ○ It is rented from a private landlord
- ○ Other (e.g. I live here rent free or home comes with job)

B5. What is the dominant type of window in your home?

- ◯ Single glazed windows
- ◯ Multiple glazed windows (i.e double)

B6. How many *separate* rooms does the property have - Only count rooms that can be closed off/separated by a door. Do not double count rooms with multiple function.

- ◯ Number of bedrooms ________________________________
- ◯ Number of bathrooms ________________________________
- ◯ Number of living/reception rooms ________________________________
- ◯ Number of other rooms ________________________________

B7. Is this home carpeted?

- ◯ Yes, totally carpeted
- ◯ Yes, partially carpeted
- ◯ No, not carpeted

Display This Question:

If B7. Is this home carpeted? = Yes, totally carpeted

And B7. Is this home carpeted? = Yes, partially carpeted

B8. How many rooms are carpeted?

____

B9. Do you have any of the following:

	Yes	No
Curtains	○	○
Blinds	○	○
Curtains and blinds	○	○

Display This Question:

If B9. Do you have any of the following: = Curtains [Yes]

And B9. Do you have any of the following: = Blinds [Yes]

And B9. Do you have any of the following: = Curtains and blinds [Yes]

B10. How many rooms have curtains and or blinds?

____

B11. Are there any rugs?

○ Yes

○ No

Display This Question:

If B11. Are there any rugs? = Yes

B12. How many rooms have rugs?

____

B13. What is your main extraction method in the **kitchen**?

- ☐ Window / door
- ☐ Extractor fan to outside
- ☐ Recirculating extractor fan
- ☐ I don't use any extraction method

B14. What is your main extraction method in the **bathroom/toilet**?

- ☐ Window
- ☐ Extractor fan
- ☐ I don't use any extractor method

B15. Are you aware of any signs of mould and or damp in your home?

- ○ Yes
- ○ No

Display This Question:

If B15. Are you aware of any signs of mould and or damp in your home? = Yes

B16. Where?

____

B17. What do you use for heating your home?   (Tick all that apply)

- ☐ Gas central heating
- ☐ Oil central heating
- ☐ Night storage heaters
- ☐ Fixed room fires or heaters
- ☐ Open fires or stoves (solid fuel / wood / coal)
- ☐ Portable heaters: Electric, Bottled gas/paraffin, Oil-filled
- ☐ Other ____

Display This Question:

If B17. What do you use for heating your home? (Tick all that apply) = Portable heaters: Electric, Bottled gas/paraffin, Oil-filled

B18. Which type of portable heater? (Tick all that apply)

- ☐ Bottled gas
- ☐ Parafin
- ☐ Oiled filled
- ☐ Other ____

B19. What fuel do you use for cooking? (Tick all that apply)

- ☐ Gas mains
- ☐ Gads bottles
- ☐ Electricity
- ☐ Other ________________________________

B20. Does your household use non-stick cookware?

- ◯ Yes, every day
- ◯ Yes, multiple times a week
- ◯ Yes, once or twice a week
- ◯ Yes, less than once a week
- ◯ No, I don't use non-stick pans
- ◯ I don't know

Display This Question:

If B20. Does your household use non-stick cookware? = Yes, every day

And B20. Does your household use non-stick cookware? = Yes, multiple times a week

And B20. Does your household use non-stick cookware? = Yes, once or twice a week

And B20. Does your household use non-stick cookware? = Yes, less than once a week

B21. How old is your non-stick cookware?

- ◯ Under 6 months
- ◯ Between 6 months- 1 year
- ◯ More than 1 year

B22. How many people in your household use personal care spray products daily? (e.g. hair spray or deodorant spray)

____

B23. What type of materials are your families’ clothes made of mostly?

- ◯ Mainly synthetic fibres
- ◯ A mix of synthetic and natural fibres
- ◯ Mainly natural fibres
- ◯ I don't know

B24. Do your household use or burn any of the following home fragrances? How many times in a week do you use this home fragrance on average? (Tick all that apply)

- ☐ No, we don’t burn home fragrances
- ☐ Candles ____
- ☐ Oud ____
- ☐ Uunsi /dabqaad/incense burner ______________________________
- ☐ Incense Sticks ____
- ☐ Reed Diffusers ____
- ☐ Oil Diffusers ____
- ☐ Air Fresheners (including plug in) ___________________________
- ☐ Other. please specify ____

B25. Are there any pets in your household?

- ○ Yes
- ○ No

Display This Question:

If B25. Are there any pets in your household? = Yes

B26. What type of pet(s) do you own and how many? (Please select all that apply)

- ☐ Dog ______________________________
- ☐ Cat ______________________________
- ☐ Bird ______________________________
- ☐ Fish ______________________________
- ☐ Hamster/Guinea Pig/ Gerbil ______________________________
- ☐ Rabbit ______________________________
- ☐ Ferret ______________________________
- ☐ Turtle or tortoise ______________________________
- ☐ Other, please specify ______________________________

B27. How often does someone smoke cigarettes, cigar or pipes **inside this home**? (please exclude e-cigarettes)

- ○ Every day
- ○ Multiple times a week
- ○ Once or twice a week
- ○ Less than once a week
- ○ Never
- ○ I don't know

B28.How often does someone use e-cigarettes (vaping) **inside this home**?

- ○ Every day
- ○ Multiple times a week
- ○ Once or twice a week
- ○ Less than once a week
- ○ Never
- ○ I don't know

B29. How often does someone smoke cigarettes, cigar or pipes **within close vicinity to this home** (e.g., courtyard, with window open, stairwell)? (please exclude e-cigarettes)

- ○ Every day
- ○ Multiple times a week
- ○ Once or twice a week
- ○ Less than once a week
- ○ Never
- ○ I don't know

B30. How often does someone use e-cigarettes (vaping) **within close vicinity to this home** (e.g., courtyard, with window open, stairwell)?

- ○ Every day
- ○ Multiple times a week
- ○ Once or twice a week
- ○ Less than once a week
- ○ Never
- ○ I don't know

B31. Is this home normally occupied during weekdays between 9am – 3pm?

- ○ No
- ○ Yes

B.32 Researcher – please take note of current meter usage for:

- ☐ Gas ______________________________
- ☐ Electricity ______________________________
- ☐ Meter access restricted/ House holder doesn't know where meter is

Section C) About your cleaning practices

C1. How often does your household use cleaning spray products?

- ○ Every day
- ○ Multiple times a week
- ○ Once or twice a week
- ○ Less than once a week
- ○ Never

C2. How often do you (or someone else in the household) dust your home?

- ○ Every day
- ○ Multiple times a week
- ○ Once or twice a week
- ○ Less than once a week
- ○ Never

C3. Which is the main method used for dusting? (Please tick one only)

- ○ Wet wipe
- ○ Dry wipe
- ○ Brushing off/using a feather duster
- ○ Using a microfiber duster
- ○ Hoover/vacuum cleaner
- ○ I don’t dust my home

C4. How often is your home vacuumed?

- ○ Every day
- ○ Multiple times a week
- ○ Once or twice a week
- ○ Less than once a week
- ○ Never

C5. Do you do laundry in the home?

- ○ Yes, washing machine only
- ○ Yes, washing machine and tumble dryer
- ○ Yes, other
- ○ No
- ○

C6. In which room? ____

C7. How often does your household do laundry in the home?

- ○ Every day
- ○ Multiple times a week
- ○ Once or twice a week
- ○ Less than once a week
- ○ Never

C8. How do you normally dry your clothes?

- ○ Tumble dryer
- ○ Rack
- ○ Tumble dryer and Rack

C9. Where do you normally dry your clothes? (Please tick all that apply)

- ☐ In the kitchen
- ☐ In the living/dining room
- ☐ In the bathroom
- ☐ In the bedroom
- ☐ Hallway
- ☐ Garden/balcony

Section D) Questions about the people living in your home

D1. How many people aged 18-year-old and over live in your home? (including you)

____

D2. What is the age, gender and ethnicity of the people aged 18-year-old and over living at home with you? (starting with you)

D3. How many children aged 0 to 17 live at home with you?

D4. What is the age and gender of the child/children living at home with you? Also, please tell us if they have asthma/allergies. *Please start with your youngest child first.*

D5. What is the **highest level of education** of the parents/guardians of each of the children living in the household?

D6. What is the employment status of the parents/guardian (as per response in D5 above) of each of the children living in the household?

• General Household Questionnaire 2 (Visit 2 and 4)

This questionnaire should take about 10 minutes to complete.

Consent: I have understood the information provided in the Participant Information Sheet and I agree to take part in this survey

○ Yes

○ No

Home ID (you can find this on your magnet) ____________________________

Date____________________________

Section A) About your Home (in the last 4 weeks)

A1. Over the past 4 weeks, how often did you or someone else in your household open the windows:

	Every day or more often	Five to six days a week	Three to four days a week	Once or twice a week	Less than once a week
In the Kitchen	○	○	○	○	○
In the bedrooms	○	○	○	○	○
In the living room	○	○	○	○	○

A2. Do you have an air purifier (including central filtration system) in your home?

○ Yes

○ No

A3. If you do, how often did you use the air purifier over the last 4 weeks? (skip if not)

- ○ Every day or more often
- ○ Five to six days a week
- ○ Three to four days a week
- ○ Once or twice a week
- ○ Less than once a week

A4. **Regarding the heating in your home.** On a scale of 1 to 5, over the last 4 weeks how did you set the temperature of your home? 1 being cold, 5 being hot. (If you left it off completely, please select 1).

- ○ 1
- ○ 2
- ○ 3
- ○ 4
- ○ 5

A5. **Over the past 4 weeks,** how often did you use the following cooking methods?

	Every day or more often	Five to six days a week	Three to four days a week	Once or twice a week	Less than once a week
Frying	○	○	○	○	○
Boiling (including kettle)	○	○	○	○	○
Baking (use oven)	○	○	○	○	○
Grilling	○	○	○	○	○
Toasting	○	○	○	○	○
Steaming	○	○	○	○	○
Air frying	○	○	○	○	○
Microwave	○	○	○	○	○

A6. Is there anything else you think we should know? (Something that happened over the last 4 weeks that could have affected the quality of the air in your home. e.g. construction works, guests visiting, additional smoking, celebrations or festivities...)

____

A7. (Visit 2 only) What activities from the educational resources (WellHome rucksack) did your children like the most, and why? Please let us know if you have any suggestions for improvements.

____

- ## General Household Questionnaire 3 (Visit 3)

This questionnaire should take about 10 minutes to complete.

Consent: I have understood the information provided in the Participant Information Sheet and I agree to take part in this survey

- ○ Yes
- ○ No

Home ID ____________________________**Date**_________________________

SECTION A) Questions about the last 3 - 6 months (since last visit)

A1. Has anything of the following occurred in your household during the last six months?

- ○ New family member
- ○ New pet
- ○ New asthma or allergy diagnosis
- ○ Started using an air purifier
- ○ Started using a kitchen or bathroom extractor
- ○ Carried out improvement works in my home
- ○ Changed the fuel I used for cooking
- ○ Change how I heat my home
- ○ New large household appliance (e.g. fridge, freezer, cooker etc.)
- ○ New laundry appliances (e.g. washing machine, dryer etc.)
- ○ New large piece of soft furniture, like sofa, bed or armchair
- ○ Nothing has changed

A2. If selected any of the above, please specify here:

____

A3. Is there anything else you think we should know? (Something that happened over the last 3-6 months that could have affected the quality of the air in your home. e.g., construction works/repairs)

____
