# Supplementary material for: West London Healthy Home and Environment (WellHome) Study: Protocol for a Community-Based Study Investigating Exposures Across the Indoor-Outdoor Air Pollution Continuum in Urban Communities
Source: Int J Environ Res Public Health. 2025 Feb 10;22(2):249. doi: 10.3390/ijerph22020249 (PMC11855092; doi:10.3390/ijerph22020249)
Supplement: Supplementary file 1 [file ijerph-22-00249-s001.zip › Supplementary File 2.pdf]

My activity of day 1

Date: ____________ Day of week : ____________

Is this a school day? ☐ Yes ☐ No

Time	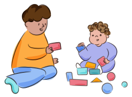 I'm at home.	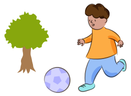 I'm outdoors, but not travelling.	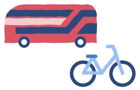 I'm travelling.	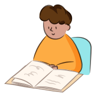 I'm indoors but not at home.
0:00 am				
0:30 am				
1:00 am				
1:30 am				
2:00 am				
2:30 am				
3:00 am				
3:30 am				
4:00 am				
4:30 am				
5:00 am				
5:30 am				
6:00 am				
6:30 am				
7:00 am				
7:30 am				
8:00 am				
8:30 am				
9:00 am				
9:30 am				
10:00 am				
10:30 am				
11:00 am				
11:30 am				
12:00 pm				
12:30 pm				
1:00 pm				
1:30 pm				
2:00 pm				
2:30 pm				
3:00 pm				
3:30 pm				

My daily activity and health diary for WellHome

My name:

My age:

I have: ☐ Asthma ☐ Allergies ☐ None of the above

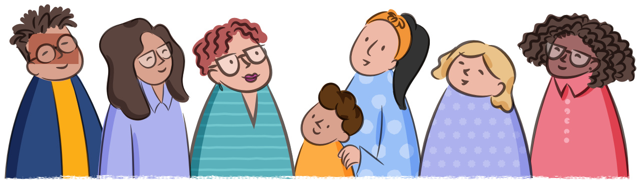

WellHome
West London Healthy Home and Environment Study

Child ID: ____________ Installation date: ____________

Home ID: ____________ Collection date: ____________

My activity of day 2

Date: ____________ Day of week : ____________

Is this a school day? ☐ Yes ☐ No

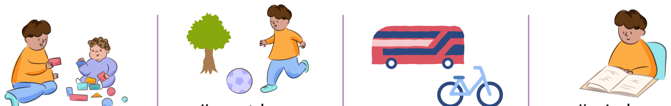

Time	I'm at home.	I'm outdoors, but not travelling.	I'm travelling.	I'm indoors but not at home.
0:00 am				
0:30 am				
1:00 am				
1:30 am				
2:00 am				
2:30 am				
3:00 am				
3:30 am				
4:00 am				
4:30 am				
5:00 am				
5:30 am				
6:00 am				
6:30 am				
7:00 am				
7:30 am				
8:00 am				
8:30 am				
9:00 am				
9:30 am				
10:00 am				
10:30 am				
11:00 am				
11:30 am				
12:00 pm				
12:30 pm				
1:00 pm				
1:30 pm				
2:00 pm				
2:30 pm				
3:00 pm				
3:30 pm				

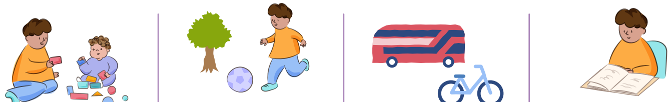

Time	I'm at home.	I'm outdoors, but not travelling.	I'm travelling.	I'm indoors but not at home.
4:00 pm				
4:30 pm				
5:00 pm				
5:30 pm				
6:00 pm				
6:30 pm				
7:00 pm				
7:30 pm				
8:00 pm				
8:30 pm				
9:00 pm				
9:30 pm				
10:00 pm				
10:30 pm				
11:00 pm				
11:30 pm				

Symptoms self-check:

Have you experienced any of the following today?

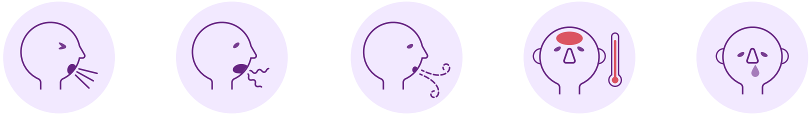

**Cough**	**Wheezing or whistling**	**Shortness of breath or Asthma**	**Fever**	**Stuffy nose**
☐ Yes ☐ No	☐ Yes ☐ No	☐ Yes ☐ No	☐ Yes ☐ No	☐ Yes ☐ No

Did you use your blue asthma inhaler?

☐ Yes ☐ No

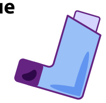

If yes, how many puffs today?

____

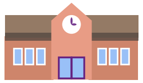

If this was a school day, were you absent due to your asthma symptoms?

☐ Yes ☐ No

Congratulations!

You completed your activity journal of the day.
Give yourself a reward sticker!

Reward sticker here.

Time	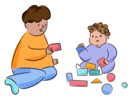 I'm at home.	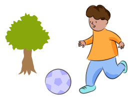 I'm outdoors, but not travelling.	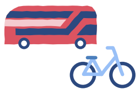 I'm travelling.	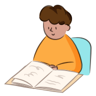 I'm indoors but not at home.
4:00 pm				
4:30 pm				
5:00 pm				
5:30 pm				
6:00 pm				
6:30 pm				
7:00 pm				
7:30 pm				
8:00 pm				
8:30 pm				
9:00 pm				
9:30 pm				
10:00 pm				
10:30 pm				
11:00 pm				
11:30 pm				

Symptoms self-check:

Have you experienced any of the following today?

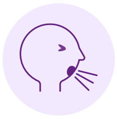

Cough

☐ Yes ☐ No

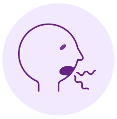

Wheezing or whistling

☐ Yes ☐ No

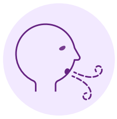

Shortness of breath or Asthma

☐ Yes ☐ No

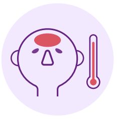

Fever

☐ Yes ☐ No

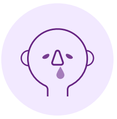

Stuffy nose

☐ Yes ☐ No

Did you use your blue asthma inhaler?

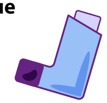

☐ Yes ☐ No

If yes, how many puffs today?

____

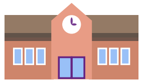

If this was a school day, were you absent due to your asthma symptoms?

☐ Yes ☐ No

Congratulations!

You completed your activity journal of the day.
Give yourself a reward sticker!

Reward sticker here.

My activity of day 3

Date: ____________ Day of week : ____________

Is this a school day? ☐ Yes ☐ No

Time	I'm at home.	I'm outdoors, but not travelling.	I'm travelling.	I'm indoors but not at home.
0:00 am				
0:30 am				
1:00 am				
1:30 am				
2:00 am				
2:30 am				
3:00 am				
3:30 am				
4:00 am				
4:30 am				
5:00 am				
5:30 am				
6:00 am				
6:30 am				
7:00 am				
7:30 am				
8:00 am				
8:30 am				
9:00 am				
9:30 am				
10:00 am				
10:30 am				
11:00 am				
11:30 am				
12:00 pm				
12:30 pm				
1:00 pm				
1:30 pm				
2:00 pm				
2:30 pm				
3:00 pm				
3:30 pm				
4:00 pm				
4:30 pm				
5:00 pm				
5:30 pm				
6:00 pm				
6:30 pm				
7:00 pm				
7:30 pm				
8:00 pm				
8:30 pm				
9:00 pm				
9:30 pm				
10:00 pm				
10:30 pm				
11:00 pm				
11:30 pm				

Symptoms self-check:

Have you experienced any of the following today?

Cough	Wheezing or whistling	Shortness of breath or Asthma	Fever	Stuffy nose
☐ Yes ☐ No	☐ Yes ☐ No	☐ Yes ☐ No	☐ Yes ☐ No	☐ Yes ☐ No

Did you use your blue asthma inhaler?

☐ Yes ☐ No

If yes, how many puffs today?

____

If this was a school day, were you absent due to your asthma symptoms?

☐ Yes ☐ No

Congratulations!

You completed your activity journal of the day.
Give yourself a reward sticker!

Reward sticker here.

My activity of day 30

Date: ____________ Day of week : ____________

Is this a school day? ☐ Yes ☐ No

Time	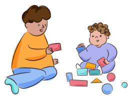 I'm at home.	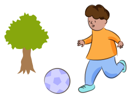 I'm outdoors, but not travelling.	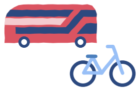 I'm travelling.	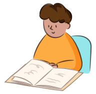 I'm indoors but not at home.
0:00 am				
0:30 am				
1:00 am				
1:30 am				
2:00 am				
2:30 am				
3:00 am				
3:30 am				
4:00 am				
4:30 am				
5:00 am				
5:30 am				
6:00 am				
6:30 am				
7:00 am				
7:30 am				
8:00 am				
8:30 am				
9:00 am				
9:30 am				
10:00 am				
10:30 am				
11:00 am				
11:30 am				
12:00 pm				
12:30 pm				
1:00 pm				
1:30 pm				
2:00 pm				
2:30 pm				
3:00 pm				
3:30 pm				

Time	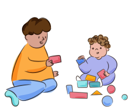 I'm at home.	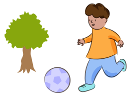 I'm outdoors, but not travelling.	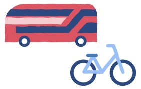 I'm travelling.	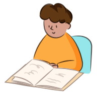 I'm indoors but not at home.
4:00 pm				
4:30 pm				
5:00 pm				
5:30 pm				
6:00 pm				
6:30 pm				
7:00 pm				
7:30 pm				
8:00 pm				
8:30 pm				
9:00 pm				
9:30 pm				
10:00 pm				
10:30 pm				
11:00 pm				
11:30 pm				

Symptoms self-check:

Have you experienced any of the following today?

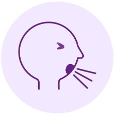

Cough

☐ Yes ☐ No

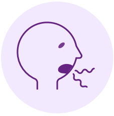

Wheezing or whistling

☐ Yes ☐ No

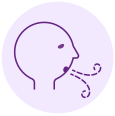

Shortness of breath or Asthma

☐ Yes ☐ No

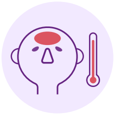

Fever

☐ Yes ☐ No

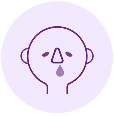

Stuffy nose

☐ Yes ☐ No

Did you use your blue asthma inhaler?

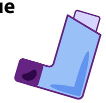

☐ Yes ☐ No

If yes, how many puffs today?

____

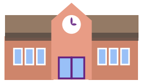

If this was a school day, were you absent due to your asthma symptoms?

☐ Yes ☐ No

Congratulations!

You completed your activity journal of the day.
Give yourself a reward sticker!

Reward sticker here.

My activity of day 4

Date: ____________ Day of week : ____________

Is this a school day? ☐ Yes ☐ No

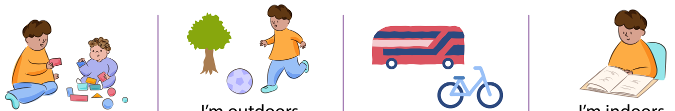

Time	I'm at home.	I'm outdoors, but not travelling.	I'm travelling.	I'm indoors but not at home.
0:00 am				
0:30 am				
1:00 am				
1:30 am				
2:00 am				
2:30 am				
3:00 am				
3:30 am				
4:00 am				
4:30 am				
5:00 am				
5:30 am				
6:00 am				
6:30 am				
7:00 am				
7:30 am				
8:00 am				
8:30 am				
9:00 am				
9:30 am				
10:00 am				
10:30 am				
11:00 am				
11:30 am				
12:00 pm				
12:30 pm				
1:00 pm				
1:30 pm				
2:00 pm				
2:30 pm				
3:00 pm				
3:30 pm				

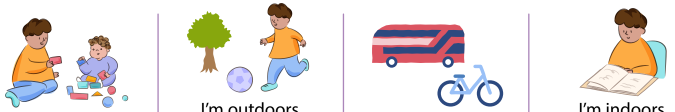

Time	I'm at home.	I'm outdoors, but not travelling.	I'm travelling.	I'm indoors but not at home.
4:00 pm				
4:30 pm				
5:00 pm				
5:30 pm				
6:00 pm				
6:30 pm				
7:00 pm				
7:30 pm				
8:00 pm				
8:30 pm				
9:00 pm				
9:30 pm				
10:00 pm				
10:30 pm				
11:00 pm				
11:30 pm				

Symptoms self-check:

Have you experienced any of the following today?

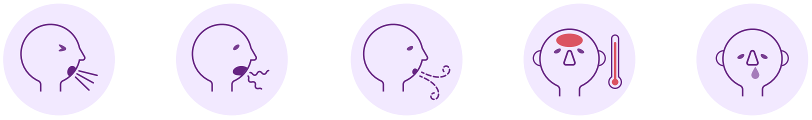

Cough	Wheezing or whistling	Shortness of breath or Asthma	Fever	Stuffy nose
☐ Yes ☐ No	☐ Yes ☐ No	☐ Yes ☐ No	☐ Yes ☐ No	☐ Yes ☐ No

Did you use your blue asthma inhaler?

☐ Yes ☐ No

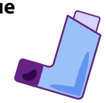

If yes, how many puffs today?

____

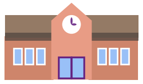

If this was a school day, were you absent due to your asthma symptoms?

☐ Yes ☐ No

Congratulations!

You completed your activity journal of the day.
Give yourself a reward sticker!

Reward sticker here.

My activity of day 29

Date: __________ Day of week : __________

Is this a school day? ☐ Yes ☐ No

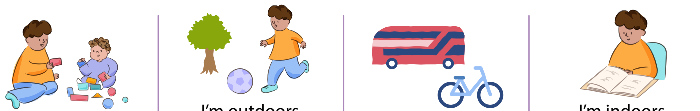

Time	I'm at home.	I'm outdoors, but not travelling.	I'm travelling.	I'm indoors but not at home.
0:00 am				
0:30 am				
1:00 am				
1:30 am				
2:00 am				
2:30 am				
3:00 am				
3:30 am				
4:00 am				
4:30 am				
5:00 am				
5:30 am				
6:00 am				
6:30 am				
7:00 am				
7:30 am				
8:00 am				
8:30 am				
9:00 am				
9:30 am				
10:00 am				
10:30 am				
11:00 am				
11:30 am				
12:00 pm				
12:30 pm				
1:00 pm				
1:30 pm				
2:00 pm				
2:30 pm				
3:00 pm				
3:30 pm				

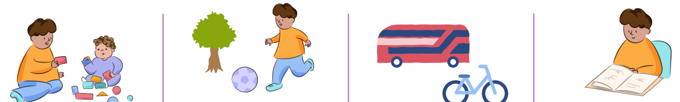

Time	I'm at home.	I'm outdoors, but not travelling.	I'm travelling.	I'm indoors but not at home.
4:00 pm				
4:30 pm				
5:00 pm				
5:30 pm				
6:00 pm				
6:30 pm				
7:00 pm				
7:30 pm				
8:00 pm				
8:30 pm				
9:00 pm				
9:30 pm				
10:00 pm				
10:30 pm				
11:00 pm				
11:30 pm				

Symptoms self-check:

Have you experienced any of the following today?

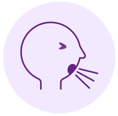

Cough

☐ Yes ☐ No

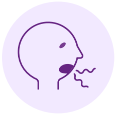

Wheezing or whistling

☐ Yes ☐ No

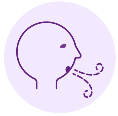

Shortness of breath or Asthma

☐ Yes ☐ No

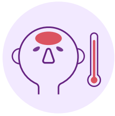

Fever

☐ Yes ☐ No

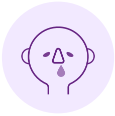

Stuffy nose

☐ Yes ☐ No

Did you use your blue asthma inhaler?

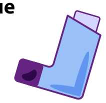

☐ Yes ☐ No

If yes, how many puffs today?

____

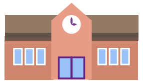

If this was a school day, were you absent due to your asthma symptoms?

☐ Yes ☐ No

Congratulations!

You completed your activity journal of the day.
Give yourself a reward sticker!

Reward sticker here.

My activity of day 5

Date: ____________ Day of week : ____________

Is this a school day? ☐ Yes ☐ No

Time	I'm at home.	I'm outdoors, but not travelling.	I'm travelling.	I'm indoors but not at home.
0:00 am				
0:30 am				
1:00 am				
1:30 am				
2:00 am				
2:30 am				
3:00 am				
3:30 am				
4:00 am				
4:30 am				
5:00 am				
5:30 am				
6:00 am				
6:30 am				
7:00 am				
7:30 am				
8:00 am				
8:30 am				
9:00 am				
9:30 am				
10:00 am				
10:30 am				
11:00 am				
11:30 am				
12:00 pm				
12:30 pm				
1:00 pm				
1:30 pm				
2:00 pm				
2:30 pm				
3:00 pm				
3:30 pm				

Time	I'm at home.	I'm outdoors, but not travelling.	I'm travelling.	I'm indoors but not at home.
4:00 pm				
4:30 pm				
5:00 pm				
5:30 pm				
6:00 pm				
6:30 pm				
7:00 pm				
7:30 pm				
8:00 pm				
8:30 pm				
9:00 pm				
9:30 pm				
10:00 pm				
10:30 pm				
11:00 pm				
11:30 pm				

Symptoms self-check:

Have you experienced any of the following today?

Cough	Wheezing or whistling	Shortness of breath or Asthma	Fever	Stuffy nose
☐ Yes ☐ No	☐ Yes ☐ No	☐ Yes ☐ No	☐ Yes ☐ No	☐ Yes ☐ No

Did you use your blue asthma inhaler?

☐ Yes ☐ No

If yes, how many puffs today?

____

If this was a school day, were you absent due to your asthma symptoms?

☐ Yes ☐ No

Congratulations!

You completed your activity journal of the day.
Give yourself a reward sticker!

Reward sticker here.

My activity of day 28

Date: ____________ Day of week : ____________

Is this a school day? ☐ Yes ☐ No

Time	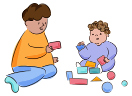 I'm at home.	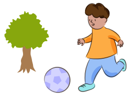 I'm outdoors, but not travelling.	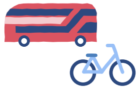 I'm travelling.	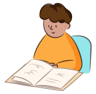 I'm indoors but not at home.
0:00 am				
0:30 am				
1:00 am				
1:30 am				
2:00 am				
2:30 am				
3:00 am				
3:30 am				
4:00 am				
4:30 am				
5:00 am				
5:30 am				
6:00 am				
6:30 am				
7:00 am				
7:30 am				
8:00 am				
8:30 am				
9:00 am				
9:30 am				
10:00 am				
10:30 am				
11:00 am				
11:30 am				
12:00 pm				
12:30 pm				
1:00 pm				
1:30 pm				
2:00 pm				
2:30 pm				
3:00 pm				
3:30 pm				

Time	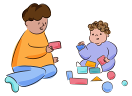 I'm at home.	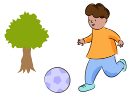 I'm outdoors, but not travelling.	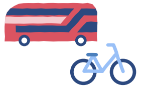 I'm travelling.	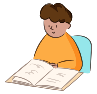 I'm indoors but not at home.
4:00 pm				
4:30 pm				
5:00 pm				
5:30 pm				
6:00 pm				
6:30 pm				
7:00 pm				
7:30 pm				
8:00 pm				
8:30 pm				
9:00 pm				
9:30 pm				
10:00 pm				
10:30 pm				
11:00 pm				
11:30 pm				

Symptoms self-check:

Have you experienced any of the following today?

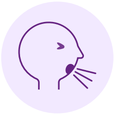

Cough

☐ Yes ☐ No

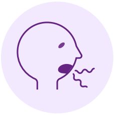

Wheezing or whistling

☐ Yes ☐ No

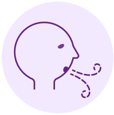

Shortness of breath or Asthma

☐ Yes ☐ No

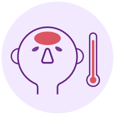

Fever

☐ Yes ☐ No

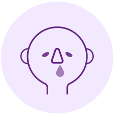

Stuffy nose

☐ Yes ☐ No

Did you use your blue asthma inhaler?

☐ Yes ☐ No

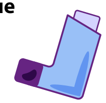

If yes, how many puffs today?

____

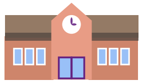

If this was a school day, were you absent due to your asthma symptoms?

☐ Yes ☐ No

Congratulations!

You completed your activity journal of the day.
Give yourself a reward sticker!

Reward sticker here.

My activity of day 6

Date: __________ Day of week : __________

Is this a school day? ☐ Yes ☐ No

Time	I'm at home.	I'm outdoors, but not travelling.	I'm travelling.	I'm indoors but not at home.
0:00 am				
0:30 am				
1:00 am				
1:30 am				
2:00 am				
2:30 am				
3:00 am				
3:30 am				
4:00 am				
4:30 am				
5:00 am				
5:30 am				
6:00 am				
6:30 am				
7:00 am				
7:30 am				
8:00 am				
8:30 am				
9:00 am				
9:30 am				
10:00 am				
10:30 am				
11:00 am				
11:30 am				
12:00 pm				
12:30 pm				
1:00 pm				
1:30 pm				
2:00 pm				
2:30 pm				
3:00 pm				
3:30 pm				

Time	I'm at home.	I'm outdoors, but not travelling.	I'm travelling.	I'm indoors but not at home.
4:00 pm				
4:30 pm				
5:00 pm				
5:30 pm				
6:00 pm				
6:30 pm				
7:00 pm				
7:30 pm				
8:00 pm				
8:30 pm				
9:00 pm				
9:30 pm				
10:00 pm				
10:30 pm				
11:00 pm				
11:30 pm				

Symptoms self-check:

Have you experienced any of the following today?

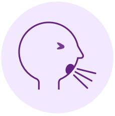

Cough

☐ Yes ☐ No

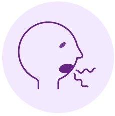

Wheezing or whistling

☐ Yes ☐ No

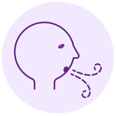

Shortness of breath or Asthma

☐ Yes ☐ No

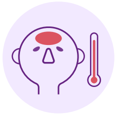

Fever

☐ Yes ☐ No

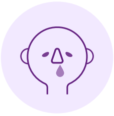

Stuffy nose

☐ Yes ☐ No

Did you use your blue asthma inhaler?

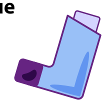

☐ Yes ☐ No

If yes, how many puffs today?

____

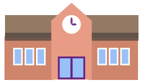

If this was a school day, were you absent due to your asthma symptoms?

☐ Yes ☐ No

Congratulations!

You completed your activity journal of the day.
Give yourself a reward sticker!

Reward sticker here.

My activity of day 27

Date: ___________ Day of week : ___________

Is this a school day? ☐ Yes ☐ No

Time	I'm at home.	I'm outdoors, but not travelling.	I'm travelling.	I'm indoors but not at home.
0:00 am				
0:30 am				
1:00 am				
1:30 am				
2:00 am				
2:30 am				
3:00 am				
3:30 am				
4:00 am				
4:30 am				
5:00 am				
5:30 am				
6:00 am				
6:30 am				
7:00 am				
7:30 am				
8:00 am				
8:30 am				
9:00 am				
9:30 am				
10:00 am				
10:30 am				
11:00 am				
11:30 am				
12:00 pm				
12:30 pm				
1:00 pm				
1:30 pm				
2:00 pm				
2:30 pm				
3:00 pm				
3:30 pm				

Time	I'm at home.	I'm outdoors, but not travelling.	I'm travelling.	I'm indoors but not at home.
4:00 pm				
4:30 pm				
5:00 pm				
5:30 pm				
6:00 pm				
6:30 pm				
7:00 pm				
7:30 pm				
8:00 pm				
8:30 pm				
9:00 pm				
9:30 pm				
10:00 pm				
10:30 pm				
11:00 pm				
11:30 pm				

Symptoms self-check:

Have you experienced any of the following today?

Cough	Wheezing or whistling	Shortness of breath or Asthma	Fever	Stuffy nose
☐ Yes ☐ No	☐ Yes ☐ No	☐ Yes ☐ No	☐ Yes ☐ No	☐ Yes ☐ No

Did you use your blue asthma inhaler?

☐ Yes ☐ No

If yes, how many puffs today?

____

If this was a school day, were you absent due to your asthma symptoms?

☐ Yes ☐ No

Congratulations!

You completed your activity journal of the day.
Give yourself a reward sticker!

Reward sticker here.

My activity of day 7

Date: ____________ Day of week : ____________

Is this a school day? ☐ Yes ☐ No

Time	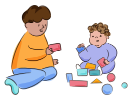 I'm at home.	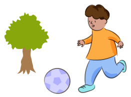 I'm outdoors, but not travelling.	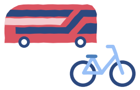 I'm travelling.	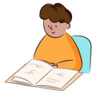 I'm indoors but not at home.
0:00 am				
0:30 am				
1:00 am				
1:30 am				
2:00 am				
2:30 am				
3:00 am				
3:30 am				
4:00 am				
4:30 am				
5:00 am				
5:30 am				
6:00 am				
6:30 am				
7:00 am				
7:30 am				
8:00 am				
8:30 am				
9:00 am				
9:30 am				
10:00 am				
10:30 am				
11:00 am				
11:30 am				
12:00 pm				
12:30 pm				
1:00 pm				
1:30 pm				
2:00 pm				
2:30 pm				
3:00 pm				
3:30 pm				

Time	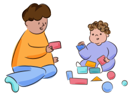 I'm at home.	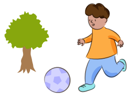 I'm outdoors, but not travelling.	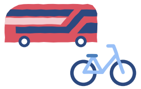 I'm travelling.	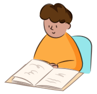 I'm indoors but not at home.
4:00 pm				
4:30 pm				
5:00 pm				
5:30 pm				
6:00 pm				
6:30 pm				
7:00 pm				
7:30 pm				
8:00 pm				
8:30 pm				
9:00 pm				
9:30 pm				
10:00 pm				
10:30 pm				
11:00 pm				
11:30 pm				

Symptoms self-check:

Have you experienced any of the following today?

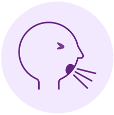

Cough

☐ Yes ☐ No

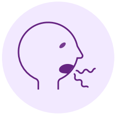

Wheezing or whistling

☐ Yes ☐ No

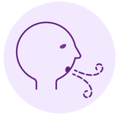

Shortness of breath or Asthma

☐ Yes ☐ No

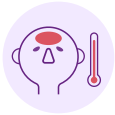

Fever

☐ Yes ☐ No

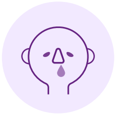

Stuffy nose

☐ Yes ☐ No

Did you use your blue asthma inhaler?

☐ Yes ☐ No

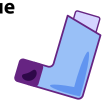

If yes, how many puffs today?

____

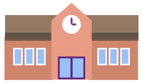

If this was a school day, were you absent due to your asthma symptoms?

☐ Yes ☐ No

Congratulations!

You completed your activity journal of the day.
Give yourself a reward sticker!

Reward sticker here.

My activity of day 26

Date: ____________ Day of week : ____________

Is this a school day? ☐ Yes ☐ No

Time	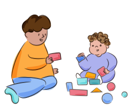 I'm at home.	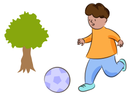 I'm outdoors, but not travelling.	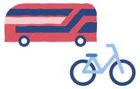 I'm travelling.	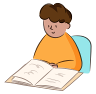 I'm indoors but not at home.
0:00 am				
0:30 am				
1:00 am				
1:30 am				
2:00 am				
2:30 am				
3:00 am				
3:30 am				
4:00 am				
4:30 am				
5:00 am				
5:30 am				
6:00 am				
6:30 am				
7:00 am				
7:30 am				
8:00 am				
8:30 am				
9:00 am				
9:30 am				
10:00 am				
10:30 am				
11:00 am				
11:30 am				
12:00 pm				
12:30 pm				
1:00 pm				
1:30 pm				
2:00 pm				
2:30 pm				
3:00 pm				
3:30 pm				

Time	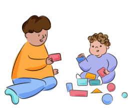 I'm at home.	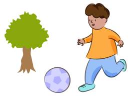 I'm outdoors, but not travelling.	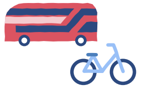 I'm travelling.	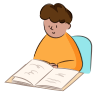 I'm indoors but not at home.
4:00 pm				
4:30 pm				
5:00 pm				
5:30 pm				
6:00 pm				
6:30 pm				
7:00 pm				
7:30 pm				
8:00 pm				
8:30 pm				
9:00 pm				
9:30 pm				
10:00 pm				
10:30 pm				
11:00 pm				
11:30 pm				

Symptoms self-check:

Have you experienced any of the following today?

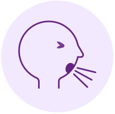

Cough

☐ Yes ☐ No

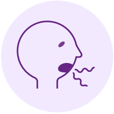

Wheezing or whistling

☐ Yes ☐ No

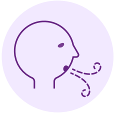

Shortness of breath or Asthma

☐ Yes ☐ No

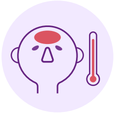

Fever

☐ Yes ☐ No

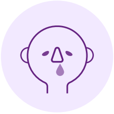

Stuffy nose

☐ Yes ☐ No

Did you use your blue asthma inhaler?

☐ Yes ☐ No

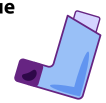

If yes, how many puffs today?

____

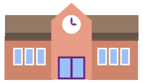

If this was a school day, were you absent due to your asthma symptoms?

☐ Yes ☐ No

Congratulations!

You completed your activity journal of the day.
Give yourself a reward sticker!

Reward sticker here.

My activity of day 8

Date: ____________ Day of week : ____________

Is this a school day? ☐ Yes ☐ No

Time	I'm at home.	I'm outdoors, but not travelling.	I'm travelling.	I'm indoors but not at home.
0:00 am				
0:30 am				
1:00 am				
1:30 am				
2:00 am				
2:30 am				
3:00 am				
3:30 am				
4:00 am				
4:30 am				
5:00 am				
5:30 am				
6:00 am				
6:30 am				
7:00 am				
7:30 am				
8:00 am				
8:30 am				
9:00 am				
9:30 am				
10:00 am				
10:30 am				
11:00 am				
11:30 am				
12:00 pm				
12:30 pm				
1:00 pm				
1:30 pm				
2:00 pm				
2:30 pm				
3:00 pm				
3:30 pm				

Time	I'm at home.	I'm outdoors, but not travelling.	I'm travelling.	I'm indoors but not at home.
4:00 pm				
4:30 pm				
5:00 pm				
5:30 pm				
6:00 pm				
6:30 pm				
7:00 pm				
7:30 pm				
8:00 pm				
8:30 pm				
9:00 pm				
9:30 pm				
10:00 pm				
10:30 pm				
11:00 pm				
11:30 pm				

Symptoms self-check:

Have you experienced any of the following today?

Cough	Wheezing or whistling	Shortness of breath or Asthma	Fever	Stuffy nose
☐ Yes ☐ No	☐ Yes ☐ No	☐ Yes ☐ No	☐ Yes ☐ No	☐ Yes ☐ No

Did you use your blue asthma inhaler?

☐ Yes ☐ No

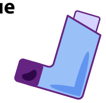

If yes, how many puffs today?

____

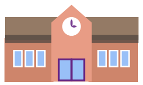

If this was a school day, were you absent due to your asthma symptoms?

☐ Yes ☐ No

Congratulations!

You completed your activity journal of the day.
Give yourself a reward sticker!

Reward sticker here.

My activity of day 25

Date: ____________ Day of week : ____________

Is this a school day? ☐ Yes ☐ No

Time	I'm at home.	I'm outdoors, but not travelling.	I'm travelling.	I'm indoors but not at home.
0:00 am				
0:30 am				
1:00 am				
1:30 am				
2:00 am				
2:30 am				
3:00 am				
3:30 am				
4:00 am				
4:30 am				
5:00 am				
5:30 am				
6:00 am				
6:30 am				
7:00 am				
7:30 am				
8:00 am				
8:30 am				
9:00 am				
9:30 am				
10:00 am				
10:30 am				
11:00 am				
11:30 am				
12:00 pm				
12:30 pm				
1:00 pm				
1:30 pm				
2:00 pm				
2:30 pm				
3:00 pm				
3:30 pm				

Time	I'm at home.	I'm outdoors, but not travelling.	I'm travelling.	I'm indoors but not at home.
4:00 pm				
4:30 pm				
5:00 pm				
5:30 pm				
6:00 pm				
6:30 pm				
7:00 pm				
7:30 pm				
8:00 pm				
8:30 pm				
9:00 pm				
9:30 pm				
10:00 pm				
10:30 pm				
11:00 pm				
11:30 pm				

Symptoms self-check:

Have you experienced any of the following today?

Cough ☐ Yes ☐ No

Wheezing or whistling ☐ Yes ☐ No

Shortness of breath or Asthma ☐ Yes ☐ No

Fever ☐ Yes ☐ No

Stuffy nose ☐ Yes ☐ No

Did you use your blue asthma inhaler?

☐ Yes ☐ No

If yes, how many puffs today?

____

If this was a school day, were you absent due to your asthma symptoms?

☐ Yes ☐ No

Congratulations!

You completed your activity journal of the day.
Give yourself a reward sticker!

Reward sticker here.

My activity of day 9

Date: ______________ Day of week : ______________

Is this a school day? ☐ Yes ☐ No

Time	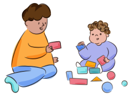 I'm at home.	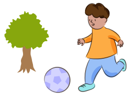 I'm outdoors, but not travelling.	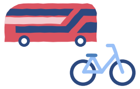 I'm travelling.	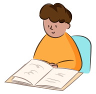 I'm indoors but not at home.
0:00 am				
0:30 am				
1:00 am				
1:30 am				
2:00 am				
2:30 am				
3:00 am				
3:30 am				
4:00 am				
4:30 am				
5:00 am				
5:30 am				
6:00 am				
6:30 am				
7:00 am				
7:30 am				
8:00 am				
8:30 am				
9:00 am				
9:30 am				
10:00 am				
10:30 am				
11:00 am				
11:30 am				
12:00 pm				
12:30 pm				
1:00 pm				
1:30 pm				
2:00 pm				
2:30 pm				
3:00 pm				
3:30 pm				

Time	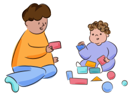 I'm at home.	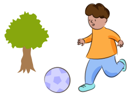 I'm outdoors, but not travelling.	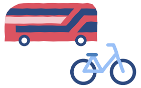 I'm travelling.	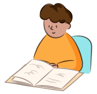 I'm indoors but not at home.
4:00 pm				
4:30 pm				
5:00 pm				
5:30 pm				
6:00 pm				
6:30 pm				
7:00 pm				
7:30 pm				
8:00 pm				
8:30 pm				
9:00 pm				
9:30 pm				
10:00 pm				
10:30 pm				
11:00 pm				
11:30 pm				

Symptoms self-check:

Have you experienced any of the following today?

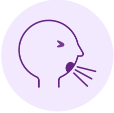

Cough

☐ Yes ☐ No

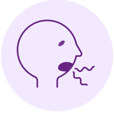

Wheezing or whistling

☐ Yes ☐ No

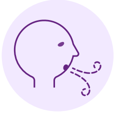

Shortness of breath or Asthma

☐ Yes ☐ No

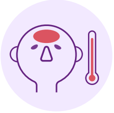

Fever

☐ Yes ☐ No

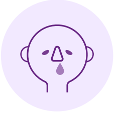

Stuffy nose

☐ Yes ☐ No

Did you use your blue asthma inhaler?

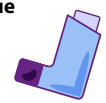

☐ Yes ☐ No

If yes, how many puffs today?

____

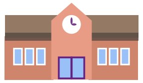

If this was a school day, were you absent due to your asthma symptoms?

☐ Yes ☐ No

Congratulations!

You completed your activity journal of the day.
Give yourself a reward sticker!

Reward sticker here.

My activity of day 24

Date: ____________ Day of week : ____________

Is this a school day? ☐ Yes ☐ No

Time	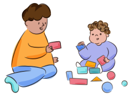 I'm at home.	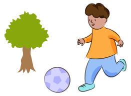 I'm outdoors, but not travelling.	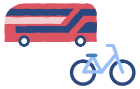 I'm travelling.	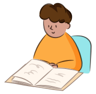 I'm indoors but not at home.
0:00 am				
0:30 am				
1:00 am				
1:30 am				
2:00 am				
2:30 am				
3:00 am				
3:30 am				
4:00 am				
4:30 am				
5:00 am				
5:30 am				
6:00 am				
6:30 am				
7:00 am				
7:30 am				
8:00 am				
8:30 am				
9:00 am				
9:30 am				
10:00 am				
10:30 am				
11:00 am				
11:30 am				
12:00 pm				
12:30 pm				
1:00 pm				
1:30 pm				
2:00 pm				
2:30 pm				
3:00 pm				
3:30 pm				

Time	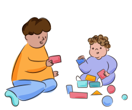 I'm at home.	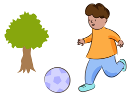 I'm outdoors, but not travelling.	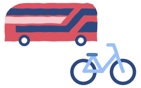 I'm travelling.	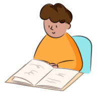 I'm indoors but not at home.
4:00 pm				
4:30 pm				
5:00 pm				
5:30 pm				
6:00 pm				
6:30 pm				
7:00 pm				
7:30 pm				
8:00 pm				
8:30 pm				
9:00 pm				
9:30 pm				
10:00 pm				
10:30 pm				
11:00 pm				
11:30 pm				

Symptoms self-check:

Have you experienced any of the following today?

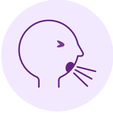

Cough

☐ Yes ☐ No

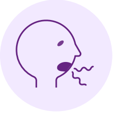

Wheezing or whistling

☐ Yes ☐ No

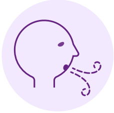

Shortness of breath or Asthma

☐ Yes ☐ No

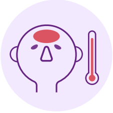

Fever

☐ Yes ☐ No

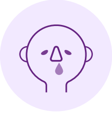

Stuffy nose

☐ Yes ☐ No

Did you use your blue asthma inhaler?

☐ Yes ☐ No

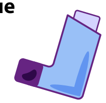

If yes, how many puffs today?

____

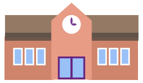

If this was a school day, were you absent due to your asthma symptoms?

☐ Yes ☐ No

Congratulations!

You completed your activity journal of the day.
Give yourself a reward sticker!

Reward sticker here.

My activity of day 10

Date: ___________ Day of week : ___________

Is this a school day? ☐ Yes ☐ No

Time	I'm at home.	I'm outdoors, but not travelling.	I'm travelling.	I'm indoors but not at home.
0:00 am				
0:30 am				
1:00 am				
1:30 am				
2:00 am				
2:30 am				
3:00 am				
3:30 am				
4:00 am				
4:30 am				
5:00 am				
5:30 am				
6:00 am				
6:30 am				
7:00 am				
7:30 am				
8:00 am				
8:30 am				
9:00 am				
9:30 am				
10:00 am				
10:30 am				
11:00 am				
11:30 am				
12:00 pm				
12:30 pm				
1:00 pm				
1:30 pm				
2:00 pm				
2:30 pm				
3:00 pm				
3:30 pm				

Time	I'm at home.	I'm outdoors, but not travelling.	I'm travelling.	I'm indoors but not at home.
4:00 pm				
4:30 pm				
5:00 pm				
5:30 pm				
6:00 pm				
6:30 pm				
7:00 pm				
7:30 pm				
8:00 pm				
8:30 pm				
9:00 pm				
9:30 pm				
10:00 pm				
10:30 pm				
11:00 pm				
11:30 pm				

Symptoms self-check:

Have you experienced any of the following today?

**Cough**	**Wheezing or whistling**	**Shortness of breath or Asthma**	**Fever**	**Stuffy nose**
☐ Yes ☐ No	☐ Yes ☐ No	☐ Yes ☐ No	☐ Yes ☐ No	☐ Yes ☐ No

Did you use your blue asthma inhaler?

☐ Yes ☐ No

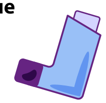

If yes, how many puffs today?

____

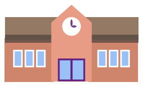

If this was a school day, were you absent due to your asthma symptoms?

☐ Yes ☐ No

Congratulations!

You completed your activity journal of the day.
Give yourself a reward sticker!

Reward sticker here.

My activity of day 23

Date: ____________ Day of week : ____________

Is this a school day? ☐ Yes ☐ No

Time	I'm at home.	I'm outdoors, but not travelling.	I'm travelling.	I'm indoors but not at home.
0:00 am				
0:30 am				
1:00 am				
1:30 am				
2:00 am				
2:30 am				
3:00 am				
3:30 am				
4:00 am				
4:30 am				
5:00 am				
5:30 am				
6:00 am				
6:30 am				
7:00 am				
7:30 am				
8:00 am				
8:30 am				
9:00 am				
9:30 am				
10:00 am				
10:30 am				
11:00 am				
11:30 am				
12:00 pm				
12:30 pm				
1:00 pm				
1:30 pm				
2:00 pm				
2:30 pm				
3:00 pm				
3:30 pm				

Time	I'm at home.	I'm outdoors, but not travelling.	I'm travelling.	I'm indoors but not at home.
4:00 pm				
4:30 pm				
5:00 pm				
5:30 pm				
6:00 pm				
6:30 pm				
7:00 pm				
7:30 pm				
8:00 pm				
8:30 pm				
9:00 pm				
9:30 pm				
10:00 pm				
10:30 pm				
11:00 pm				
11:30 pm				

Symptoms self-check:

Have you experienced any of the following today?

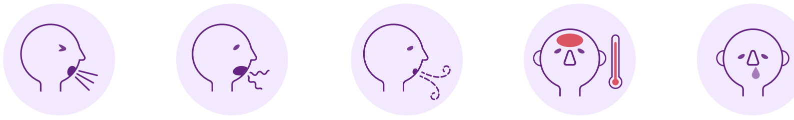

Cough	Wheezing or whistling	Shortness of breath or Asthma	Fever	Stuffy nose
☐ Yes ☐ No	☐ Yes ☐ No	☐ Yes ☐ No	☐ Yes ☐ No	☐ Yes ☐ No

Did you use your blue asthma inhaler?

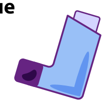

☐ Yes ☐ No

If yes, how many puffs today?

____

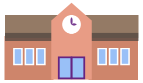

If this was a school day, were you absent due to your asthma symptoms?

☐ Yes ☐ No

Congratulations!

You completed your activity journal of the day.
Give yourself a reward sticker!

Reward sticker here.

My activity of day 11

Date: ___________ Day of week : ___________

Is this a school day? ☐ Yes ☐ No

Time	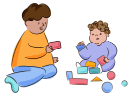 I'm at home.	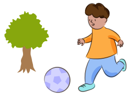 I'm outdoors, but not travelling.	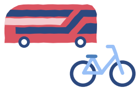 I'm travelling.	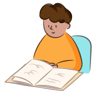 I'm indoors but not at home.
0:00 am				
0:30 am				
1:00 am				
1:30 am				
2:00 am				
2:30 am				
3:00 am				
3:30 am				
4:00 am				
4:30 am				
5:00 am				
5:30 am				
6:00 am				
6:30 am				
7:00 am				
7:30 am				
8:00 am				
8:30 am				
9:00 am				
9:30 am				
10:00 am				
10:30 am				
11:00 am				
11:30 am				
12:00 pm				
12:30 pm				
1:00 pm				
1:30 pm				
2:00 pm				
2:30 pm				
3:00 pm				
3:30 pm				

Time	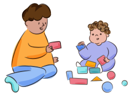 I'm at home.	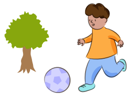 I'm outdoors, but not travelling.	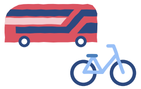 I'm travelling.	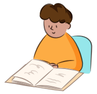 I'm indoors but not at home.
4:00 pm				
4:30 pm				
5:00 pm				
5:30 pm				
6:00 pm				
6:30 pm				
7:00 pm				
7:30 pm				
8:00 pm				
8:30 pm				
9:00 pm				
9:30 pm				
10:00 pm				
10:30 pm				
11:00 pm				
11:30 pm				

Symptoms self-check:

Have you experienced any of the following today?

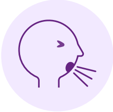

Cough

☐ Yes ☐ No

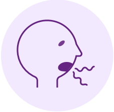

Wheezing or whistling

☐ Yes ☐ No

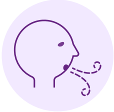

Shortness of breath or Asthma

☐ Yes ☐ No

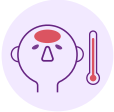

Fever

☐ Yes ☐ No

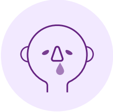

Stuffy nose

☐ Yes ☐ No

Did you use your blue asthma inhaler?

☐ Yes ☐ No

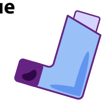

If yes, how many puffs today?

____

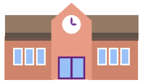

If this was a school day, were you absent due to your asthma symptoms?

☐ Yes ☐ No

Congratulations!

You completed your activity journal of the day.
Give yourself a reward sticker!

Reward sticker here.

My activity of day 22

Date: ____________ Day of week : ____________

Is this a school day? ☐ Yes ☐ No

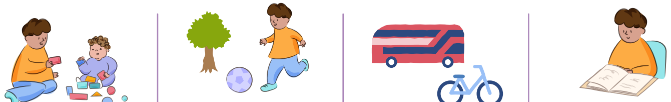

Time	I'm at home.	I'm outdoors, but not travelling.	I'm travelling.	I'm indoors but not at home.
0:00 am				
0:30 am				
1:00 am				
1:30 am				
2:00 am				
2:30 am				
3:00 am				
3:30 am				
4:00 am				
4:30 am				
5:00 am				
5:30 am				
6:00 am				
6:30 am				
7:00 am				
7:30 am				
8:00 am				
8:30 am				
9:00 am				
9:30 am				
10:00 am				
10:30 am				
11:00 am				
11:30 am				
12:00 pm				
12:30 pm				
1:00 pm				
1:30 pm				
2:00 pm				
2:30 pm				
3:00 pm				
3:30 pm				

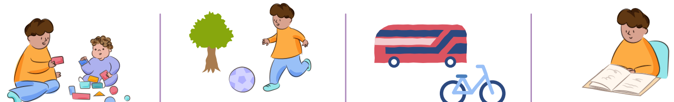

Time	I'm at home.	I'm outdoors, but not travelling.	I'm travelling.	I'm indoors but not at home.
4:00 pm				
4:30 pm				
5:00 pm				
5:30 pm				
6:00 pm				
6:30 pm				
7:00 pm				
7:30 pm				
8:00 pm				
8:30 pm				
9:00 pm				
9:30 pm				
10:00 pm				
10:30 pm				
11:00 pm				
11:30 pm				

Symptoms self-check:

Have you experienced any of the following today?

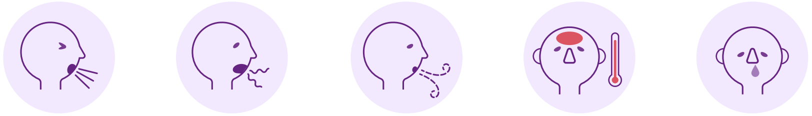

Cough ☐ Yes ☐ No

Wheezing or whistling ☐ Yes ☐ No

Shortness of breath or Asthma ☐ Yes ☐ No

Fever ☐ Yes ☐ No

Stuffy nose ☐ Yes ☐ No

Did you use your blue asthma inhaler?

☐ Yes ☐ No

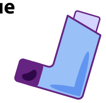

If yes, how many puffs today?

____

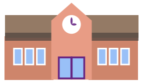

If this was a school day, were you absent due to your asthma symptoms?

☐ Yes ☐ No

Congratulations!

You completed your activity journal of the day.
Give yourself a reward sticker!

Reward sticker here.

My activity of day 12

Date: ____________ Day of week : ____________

Is this a school day? ☐ Yes ☐ No

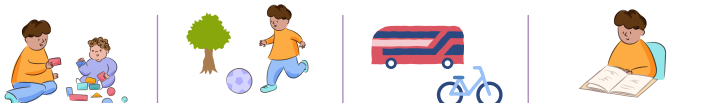

Time	I'm at home.	I'm outdoors, but not travelling.	I'm travelling.	I'm indoors but not at home.
0:00 am				
0:30 am				
1:00 am				
1:30 am				
2:00 am				
2:30 am				
3:00 am				
3:30 am				
4:00 am				
4:30 am				
5:00 am				
5:30 am				
6:00 am				
6:30 am				
7:00 am				
7:30 am				
8:00 am				
8:30 am				
9:00 am				
9:30 am				
10:00 am				
10:30 am				
11:00 am				
11:30 am				
12:00 pm				
12:30 pm				
1:00 pm				
1:30 pm				
2:00 pm				
2:30 pm				
3:00 pm				
3:30 pm				

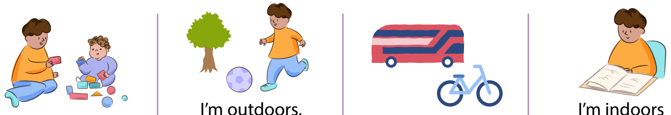

Time	I'm at home.	I'm outdoors, but not travelling.	I'm travelling.	I'm indoors but not at home.
4:00 pm				
4:30 pm				
5:00 pm				
5:30 pm				
6:00 pm				
6:30 pm				
7:00 pm				
7:30 pm				
8:00 pm				
8:30 pm				
9:00 pm				
9:30 pm				
10:00 pm				
10:30 pm				
11:00 pm				
11:30 pm				

Symptoms self-check:

Have you experienced any of the following today?

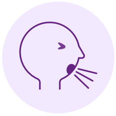

Cough ☐ Yes ☐ No

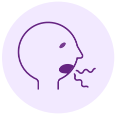

Wheezing or whistling ☐ Yes ☐ No

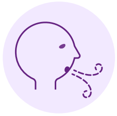

Shortness of breath or Asthma ☐ Yes ☐ No

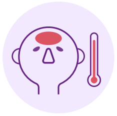

Fever ☐ Yes ☐ No

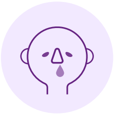

Stuffy nose ☐ Yes ☐ No

Did you use your blue asthma inhaler?

☐ Yes ☐ No

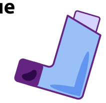

If yes, how many puffs today?

____

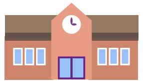

If this was a school day, were you absent due to your asthma symptoms?

☐ Yes ☐ No

Congratulations!

You completed your activity journal of the day.
Give yourself a reward sticker!

Reward sticker here.

My activity of day 21

Date: ___________ Day of week : ___________

Is this a school day? ☐ Yes ☐ No

Time	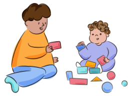 I'm at home.	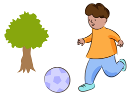 I'm outdoors, but not travelling.	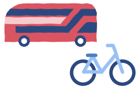 I'm travelling.	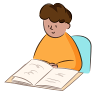 I'm indoors but not at home.
0:00 am				
0:30 am				
1:00 am				
1:30 am				
2:00 am				
2:30 am				
3:00 am				
3:30 am				
4:00 am				
4:30 am				
5:00 am				
5:30 am				
6:00 am				
6:30 am				
7:00 am				
7:30 am				
8:00 am				
8:30 am				
9:00 am				
9:30 am				
10:00 am				
10:30 am				
11:00 am				
11:30 am				
12:00 pm				
12:30 pm				
1:00 pm				
1:30 pm				
2:00 pm				
2:30 pm				
3:00 pm				
3:30 pm				

Time	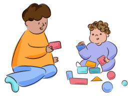 I'm at home.	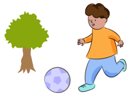 I'm outdoors, but not travelling.	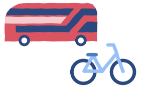 I'm travelling.	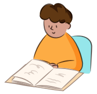 I'm indoors but not at home.
4:00 pm				
4:30 pm				
5:00 pm				
5:30 pm				
6:00 pm				
6:30 pm				
7:00 pm				
7:30 pm				
8:00 pm				
8:30 pm				
9:00 pm				
9:30 pm				
10:00 pm				
10:30 pm				
11:00 pm				
11:30 pm				

Symptoms self-check:

Have you experienced any of the following today?

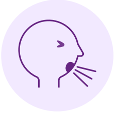
Cough
☐ Yes ☐ No

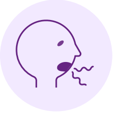
Wheezing or whistling
☐ Yes ☐ No

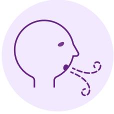
Shortness of breath or Asthma
☐ Yes ☐ No

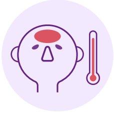
Fever
☐ Yes ☐ No

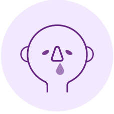
Stuffy nose
☐ Yes ☐ No

Did you use your blue asthma inhaler?

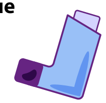

☐ Yes ☐ No

If yes, how many puffs today?

____

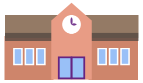

If this was a school day, were you absent due to your asthma symptoms?

☐ Yes ☐ No

Congratulations!

You completed your activity journal of the day.
Give yourself a reward sticker!

Reward sticker here.

My activity of day 13

Date: ____________ Day of week : ____________

Is this a school day? ☐ Yes ☐ No

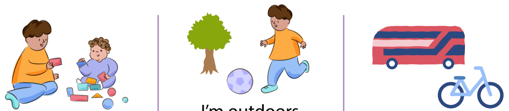

Time	I'm at home.	I'm outdoors, but not travelling.	I'm travelling.	I'm indoors but not at home.
0:00 am				
0:30 am				
1:00 am				
1:30 am				
2:00 am				
2:30 am				
3:00 am				
3:30 am				
4:00 am				
4:30 am				
5:00 am				
5:30 am				
6:00 am				
6:30 am				
7:00 am				
7:30 am				
8:00 am				
8:30 am				
9:00 am				
9:30 am				
10:00 am				
10:30 am				
11:00 am				
11:30 am				
12:00 pm				
12:30 pm				
1:00 pm				
1:30 pm				
2:00 pm				
2:30 pm				
3:00 pm				
3:30 pm				

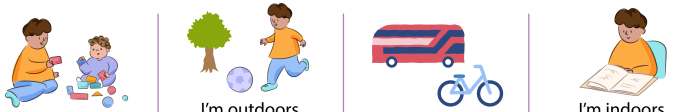

Time	I'm at home.	I'm outdoors, but not travelling.	I'm travelling.	I'm indoors but not at home.
4:00 pm				
4:30 pm				
5:00 pm				
5:30 pm				
6:00 pm				
6:30 pm				
7:00 pm				
7:30 pm				
8:00 pm				
8:30 pm				
9:00 pm				
9:30 pm				
10:00 pm				
10:30 pm				
11:00 pm				
11:30 pm				

Symptoms self-check:

Have you experienced any of the following today?

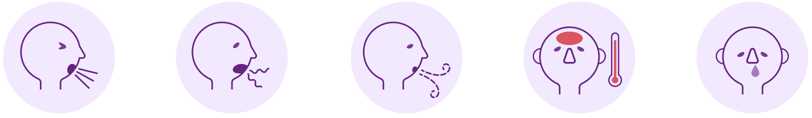

Cough	Wheezing or whistling	Shortness of breath or Asthma	Fever	Stuffy nose
☐ Yes ☐ No	☐ Yes ☐ No	☐ Yes ☐ No	☐ Yes ☐ No	☐ Yes ☐ No

Did you use your blue asthma inhaler?

☐ Yes ☐ No

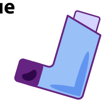

If yes, how many puffs today?

____

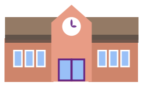

If this was a school day, were you absent due to your asthma symptoms?

☐ Yes ☐ No

Congratulations!

You completed your activity journal of the day.
Give yourself a reward sticker!

Reward sticker here.

My activity of day 20

Date: ______ Day of week : ______

Is this a school day? ☐ Yes ☐ No

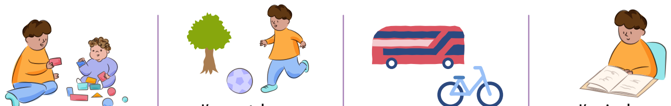

Time	I'm at home.	I'm outdoors, but not travelling.	I'm travelling.	I'm indoors but not at home.
0:00 am				
0:30 am				
1:00 am				
1:30 am				
2:00 am				
2:30 am				
3:00 am				
3:30 am				
4:00 am				
4:30 am				
5:00 am				
5:30 am				
6:00 am				
6:30 am				
7:00 am				
7:30 am				
8:00 am				
8:30 am				
9:00 am				
9:30 am				
10:00 am				
10:30 am				
11:00 am				
11:30 am				
12:00 pm				
12:30 pm				
1:00 pm				
1:30 pm				
2:00 pm				
2:30 pm				
3:00 pm				
3:30 pm				

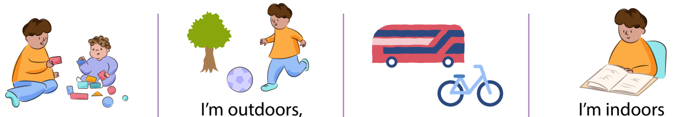

Time	I'm at home.	I'm outdoors, but not travelling.	I'm travelling.	I'm indoors but not at home.
4:00 pm				
4:30 pm				
5:00 pm				
5:30 pm				
6:00 pm				
6:30 pm				
7:00 pm				
7:30 pm				
8:00 pm				
8:30 pm				
9:00 pm				
9:30 pm				
10:00 pm				
10:30 pm				
11:00 pm				
11:30 pm				

Symptoms self-check:

Have you experienced any of the following today?

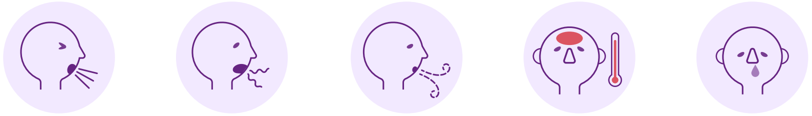

**Cough**	**Wheezing or whistling**	**Shortness of breath or Asthma**	**Fever**	**Stuffy nose**
☐ Yes ☐ No	☐ Yes ☐ No	☐ Yes ☐ No	☐ Yes ☐ No	☐ Yes ☐ No

Did you use your blue asthma inhaler?

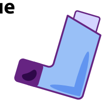

☐ Yes ☐ No

If yes, how many puffs today?

______

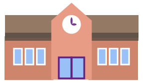

If this was a school day, were you absent due to your asthma symptoms?

☐ Yes ☐ No

Congratulations!

You completed your activity journal of the day.
Give yourself a reward sticker!

Reward sticker here.

My activity of day 14

Date: ____________ Day of week : ____________

Is this a school day? ☐ Yes ☐ No

Time	I'm at home.	I'm outdoors, but not travelling.	I'm travelling.	I'm indoors but not at home.
0:00 am				
0:30 am				
1:00 am				
1:30 am				
2:00 am				
2:30 am				
3:00 am				
3:30 am				
4:00 am				
4:30 am				
5:00 am				
5:30 am				
6:00 am				
6:30 am				
7:00 am				
7:30 am				
8:00 am				
8:30 am				
9:00 am				
9:30 am				
10:00 am				
10:30 am				
11:00 am				
11:30 am				
12:00 pm				
12:30 pm				
1:00 pm				
1:30 pm				
2:00 pm				
2:30 pm				
3:00 pm				
3:30 pm				

Time	I'm at home.	I'm outdoors, but not travelling.	I'm travelling.	I'm indoors but not at home.
4:00 pm				
4:30 pm				
5:00 pm				
5:30 pm				
6:00 pm				
6:30 pm				
7:00 pm				
7:30 pm				
8:00 pm				
8:30 pm				
9:00 pm				
9:30 pm				
10:00 pm				
10:30 pm				
11:00 pm				
11:30 pm				

Symptoms self-check:

Have you experienced any of the following today?

Cough	Wheezing or whistling	Shortness of breath or Asthma	Fever	Stuffy nose
☐ Yes ☐ No	☐ Yes ☐ No	☐ Yes ☐ No	☐ Yes ☐ No	☐ Yes ☐ No

Did you use your blue asthma inhaler?

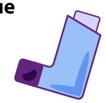

☐ Yes ☐ No

If yes, how many puffs today?

____

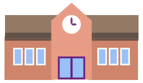

If this was a school day, were you absent due to your asthma symptoms?

☐ Yes ☐ No

Congratulations!

You completed your activity journal of the day.
Give yourself a reward sticker!

Reward sticker here.

My activity of day 19

Date: ____________ Day of week : ____________

Is this a school day? ☐ Yes ☐ No

Time	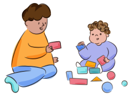 I'm at home.	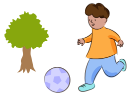 I'm outdoors, but not travelling.	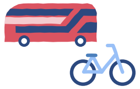 I'm travelling.	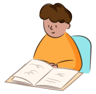 I'm indoors but not at home.
0:00 am				
0:30 am				
1:00 am				
1:30 am				
2:00 am				
2:30 am				
3:00 am				
3:30 am				
4:00 am				
4:30 am				
5:00 am				
5:30 am				
6:00 am				
6:30 am				
7:00 am				
7:30 am				
8:00 am				
8:30 am				
9:00 am				
9:30 am				
10:00 am				
10:30 am				
11:00 am				
11:30 am				
12:00 pm				
12:30 pm				
1:00 pm				
1:30 pm				
2:00 pm				
2:30 pm				
3:00 pm				
3:30 pm				

Time	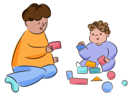 I'm at home.	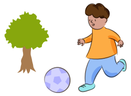 I'm outdoors, but not travelling.	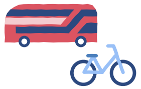 I'm travelling.	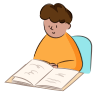 I'm indoors but not at home.
4:00 pm				
4:30 pm				
5:00 pm				
5:30 pm				
6:00 pm				
6:30 pm				
7:00 pm				
7:30 pm				
8:00 pm				
8:30 pm				
9:00 pm				
9:30 pm				
10:00 pm				
10:30 pm				
11:00 pm				
11:30 pm				

Symptoms self-check:

Have you experienced any of the following today?

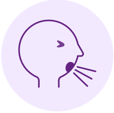

Cough

☐ Yes ☐ No

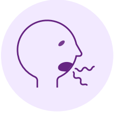

Wheezing or whistling

☐ Yes ☐ No

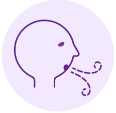

Shortness of breath or Asthma

☐ Yes ☐ No

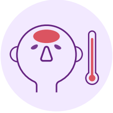

Fever

☐ Yes ☐ No

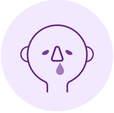

Stuffy nose

☐ Yes ☐ No

Did you use your blue asthma inhaler?

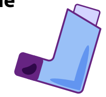

☐ Yes ☐ No

If yes, how many puffs today?

____

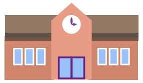

If this was a school day, were you absent due to your asthma symptoms?

☐ Yes ☐ No

Congratulations!

You completed your activity journal of the day.
Give yourself a reward sticker!

Reward sticker here.

My activity of day 15

Date: ____________ Day of week : ____________

Is this a school day? ☐ Yes ☐ No

Time	I'm at home.	I'm outdoors, but not travelling.	I'm travelling.	I'm indoors but not at home.
0:00 am				
0:30 am				
1:00 am				
1:30 am				
2:00 am				
2:30 am				
3:00 am				
3:30 am				
4:00 am				
4:30 am				
5:00 am				
5:30 am				
6:00 am				
6:30 am				
7:00 am				
7:30 am				
8:00 am				
8:30 am				
9:00 am				
9:30 am				
10:00 am				
10:30 am				
11:00 am				
11:30 am				
12:00 pm				
12:30 pm				
1:00 pm				
1:30 pm				
2:00 pm				
2:30 pm				
3:00 pm				
3:30 pm				

Time	I'm at home.	I'm outdoors, but not travelling.	I'm travelling.	I'm indoors but not at home.
4:00 pm				
4:30 pm				
5:00 pm				
5:30 pm				
6:00 pm				
6:30 pm				
7:00 pm				
7:30 pm				
8:00 pm				
8:30 pm				
9:00 pm				
9:30 pm				
10:00 pm				
10:30 pm				
11:00 pm				
11:30 pm				

Symptoms self-check:

Have you experienced any of the following today?

Cough ☐ Yes ☐ No

Wheezing or whistling ☐ Yes ☐ No

Shortness of breath or Asthma ☐ Yes ☐ No

Fever ☐ Yes ☐ No

Stuffy nose ☐ Yes ☐ No

Did you use your blue asthma inhaler?

☐ Yes ☐ No

If yes, how many puffs today?

____

If this was a school day, were you absent due to your asthma symptoms?

☐ Yes ☐ No

Congratulations!

You completed your activity journal of the day.
Give yourself a reward sticker!

Reward sticker here.

My activity of day 18

Date: ____________ Day of week : ____________

Is this a school day? ☐ Yes ☐ No

Time	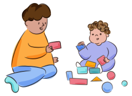 I'm at home.	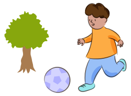 I'm outdoors, but not travelling.	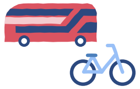 I'm travelling.	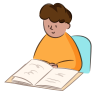 I'm indoors but not at home.
0:00 am				
0:30 am				
1:00 am				
1:30 am				
2:00 am				
2:30 am				
3:00 am				
3:30 am				
4:00 am				
4:30 am				
5:00 am				
5:30 am				
6:00 am				
6:30 am				
7:00 am				
7:30 am				
8:00 am				
8:30 am				
9:00 am				
9:30 am				
10:00 am				
10:30 am				
11:00 am				
11:30 am				
12:00 pm				
12:30 pm				
1:00 pm				
1:30 pm				
2:00 pm				
2:30 pm				
3:00 pm				
3:30 pm				

Time	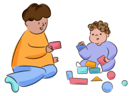 I'm at home.	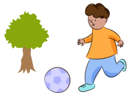 I'm outdoors, but not travelling.	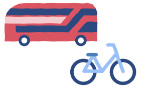 I'm travelling.	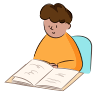 I'm indoors but not at home.
4:00 pm				
4:30 pm				
5:00 pm				
5:30 pm				
6:00 pm				
6:30 pm				
7:00 pm				
7:30 pm				
8:00 pm				
8:30 pm				
9:00 pm				
9:30 pm				
10:00 pm				
10:30 pm				
11:00 pm				
11:30 pm				

Symptoms self-check:

Have you experienced any of the following today?

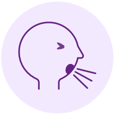

Cough

☐ Yes ☐ No

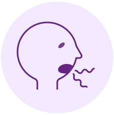

Wheezing or whistling

☐ Yes ☐ No

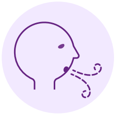

Shortness of breath or Asthma

☐ Yes ☐ No

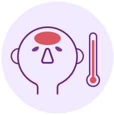

Fever

☐ Yes ☐ No

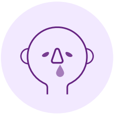

Stuffy nose

☐ Yes ☐ No

Did you use your blue asthma inhaler?

☐ Yes ☐ No

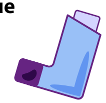

If yes, how many puffs today?

____

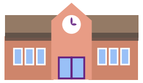

If this was a school day, were you absent due to your asthma symptoms?

☐ Yes ☐ No

Congratulations!

You completed your activity journal of the day.
Give yourself a reward sticker!

Reward sticker here.

My activity of day 16

Date: ___________ Day of week : ___________

Is this a school day? ☐ Yes ☐ No

Time	I'm at home.	I'm outdoors, but not travelling.	I'm travelling.	I'm indoors but not at home.
0:00 am				
0:30 am				
1:00 am				
1:30 am				
2:00 am				
2:30 am				
3:00 am				
3:30 am				
4:00 am				
4:30 am				
5:00 am				
5:30 am				
6:00 am				
6:30 am				
7:00 am				
7:30 am				
8:00 am				
8:30 am				
9:00 am				
9:30 am				
10:00 am				
10:30 am				
11:00 am				
11:30 am				
12:00 pm				
12:30 pm				
1:00 pm				
1:30 pm				
2:00 pm				
2:30 pm				
3:00 pm				
3:30 pm				
4:00 pm				
4:30 pm				
5:00 pm				
5:30 pm				
6:00 pm				
6:30 pm				
7:00 pm				
7:30 pm				
8:00 pm				
8:30 pm				
9:00 pm				
9:30 pm				
10:00 pm				
10:30 pm				
11:00 pm				
11:30 pm				

Symptoms self-check:

Have you experienced any of the following today?

Cough ☐ Yes ☐ No

Wheezing or whistling ☐ Yes ☐ No

Shortness of breath or Asthma ☐ Yes ☐ No

Fever ☐ Yes ☐ No

Stuffy nose ☐ Yes ☐ No

Did you use your blue asthma inhaler?

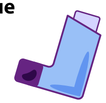

☐ Yes ☐ No

If yes, how many puffs today?

____

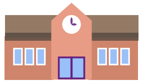

If this was a school day, were you absent due to your asthma symptoms?

☐ Yes ☐ No

Congratulations!

You completed your activity journal of the day.
Give yourself a reward sticker!

Reward sticker here.

My activity of day 17

Date: ___________ Day of week : ___________

Is this a school day? ☐ Yes ☐ No

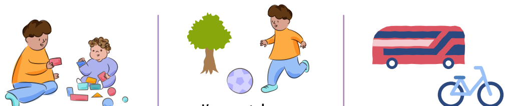

Time	I'm at home.	I'm outdoors, but not travelling.	I'm travelling.	I'm indoors but not at home.
0:00 am				
0:30 am				
1:00 am				
1:30 am				
2:00 am				
2:30 am				
3:00 am				
3:30 am				
4:00 am				
4:30 am				
5:00 am				
5:30 am				
6:00 am				
6:30 am				
7:00 am				
7:30 am				
8:00 am				
8:30 am				
9:00 am				
9:30 am				
10:00 am				
10:30 am				
11:00 am				
11:30 am				
12:00 pm				
12:30 pm				
1:00 pm				
1:30 pm				
2:00 pm				
2:30 pm				
3:00 pm				
3:30 pm				

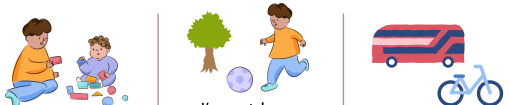

Time	I'm at home.	I'm outdoors, but not travelling.	I'm travelling.	I'm indoors but not at home.
4:00 pm				
4:30 pm				
5:00 pm				
5:30 pm				
6:00 pm				
6:30 pm				
7:00 pm				
7:30 pm				
8:00 pm				
8:30 pm				
9:00 pm				
9:30 pm				
10:00 pm				
10:30 pm				
11:00 pm				
11:30 pm				

Symptoms self-check:

Have you experienced any of the following today?

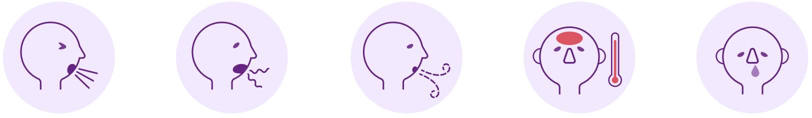

Cough	Wheezing or whistling	Shortness of breath or Asthma	Fever	Stuffy nose
☐ Yes ☐ No	☐ Yes ☐ No	☐ Yes ☐ No	☐ Yes ☐ No	☐ Yes ☐ No

Did you use your blue asthma inhaler?

☐ Yes ☐ No

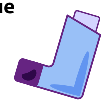

If yes, how many puffs today?

____

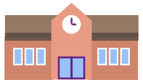

If this was a school day, were you absent due to your asthma symptoms?

☐ Yes ☐ No

Congratulations!

You completed your activity journal of the day.
Give yourself a reward sticker!

Reward sticker here.
