# Supplementary material for: West London Healthy Home and Environment (WellHome) Study: Protocol for a Community-Based Study Investigating Exposures Across the Indoor-Outdoor Air Pollution Continuum in Urban Communities
Source: Int J Environ Res Public Health. 2025 Feb 10;22(2):249. doi: 10.3390/ijerph22020249 (PMC11855092; doi:10.3390/ijerph22020249)
Supplement: Supplementary file 1 [file ijerph-22-00249-s001.zip › Supplementary File 3.pdf]

Supplementary File 3

Perceptions Questionnaire

This questionnaire should take about 15 minutes to complete.

Consent: I have understood the information provided in the Participant Information Sheet and I agree to take part in this survey

◯ Yes

◯ No

Home ID ________________ **Date** ______________

First, we would like to ask you a question about a number of things inside and outside the home. We would like to know how worried you are about them.

1. How worried, if at all, are you about the following issues at present?

	Not at all worried	Not very worried	Somewhat worried	Very worried	Extremely worried
Food poisoning	◯	◯	◯	◯	◯
Household chemicals	◯	◯	◯	◯	◯
Outdoor air pollution (air quality outside the home)	◯	◯	◯	◯	◯
Indoor air pollution (air quality inside the home)	◯	◯	◯	◯	◯
Carbon monoxide	◯	◯	◯	◯	◯
The economy	◯	◯	◯	◯	◯
Cost of living	◯	◯	◯	◯	◯

The next questions are about the quality of the air in and around your house. There are no right or wrong answers to the questions. We are interested in how you feel about indoor and outdoor air quality.

2. How do you rate the quality of the air in the following places?

	Very good	Good	Neither good nor bad	Poor	Very poor
Your home	○	○	○	○	○
Your street	○	○	○	○	○
Your neighborhood	○	○	○	○	○
London overall	○	○	○	○	○

3. How much have you thought about the following before today?

	Not at all	Very little	Some	A lot	A great deal
The sources of air pollutants outside your home	○	○	○	○	○
The sources of air pollutants inside your home	○	○	○	○	○
The levels of air pollution outside your home	○	○	○	○	○
The levels of air pollution inside your home	○	○	○	○	○
The health effects of poor outdoor air quality	○	○	○	○	○
The health effects of poor indoor air quality	○	○	○	○	○
The things you could do to reduce exposure to harmful pollutants outside your home	○	○	○	○	○
The things you could do to reduce exposure to harmful pollutants inside your home	○	○	○	○	○

4. What do you think are the most important sources of air pollution inside your home? (Tick up to five options)

- ☐ Construction/builders/road works
- ☐ Traffic
- ☐ Smoking inside the home
- ☐ Smoking within vicinity of your home
- ☐ Pets
- ☐ Furniture
- ☐ Carpet
- ☐ Cleaning products
- ☐ Personal care products (e.g., deodorant)
- ☐ Air fresheners
- ☐ Paint
- ☐ Waste bins inside the home
- ☐ Mould
- ☐ Cooking

- [ ] Fireplace/ Wood burners
- [ ] Gas/central heating
- [ ] Candles, scent sticks, etc.
- [ ] Dust
- [ ] Other (please tell us) ____________________

5. To what extent do you agree or disagree with the following statement

	Strongly agree	Somewhat agree	Neither agree nor disagree	Somewhat disagree	Strongly disagree
Poor indoor air quality is harmful to human health	○	○	○	○	○
The health consequences from poor indoor air quality can be severe	○	○	○	○	○

6.How serious do you think the health consequences of indoor air pollution/ poor indoor air quality are?

- ○ Not at all serious
- ○ Not very serious
- ○ Somewhat serious
- ○ Very serious
- ○ Extremely serious

7.To what extent do you think you and your family are personally affected by poor indoor air quality?

- ○ Not at all affected
- ○ Not very much affected
- ○ Somewhat affected
- ○ Very affected
- ○ Extremely affected

8.How likely do you think it is that your health will be affected by poor indoor air quality?

- ◯ Not at all likely
- ◯ Not very likely
- ◯ Somewhat likely
- ◯ Very likely
- ◯ Extremely likely

9.To what extent do you agree or disagree with the following statement

	Strongly agree	Somewhat agree	Neither agree nor disagree	Somewhat disagree	Strongly disagree
My health is at risk from poor indoor air quality when I am at home	◯	◯	◯	◯	◯
I feel personally at risk from poor indoor air quality	◯	◯	◯	◯	◯

10.To what extent do you feel that your own personal actions can help to improve the air quality in your home?

- ○ Not at all
- ○ Very little
- ○ Some
- ○ A lot
- ○ A great deal

11.How confident are you that you can take actions to improve the air quality in your home?

- ○ Not at all confident
- ○ Not very confident
- ○ Somewhat confident
- ○ Very confident
- ○ Extremely confident

---

12. How much control do you feel you have over the following?

	No control at all	Little control	Some control	A lot of control	Complete control
Protecting yourself and members of your household against indoor poor indoor air quality?	○	○	○	○	○
Taking action to improve the air quality in your home?	○	○	○	○	○

13. Overall, how satisfied or dissatisfied are you with the quality of the air inside your home?

- ○ Very satisfied
- ○ Fairly satisfied
- ○ Neither satisfied nor dissatisfied
- ○ Fairly dissatisfied
- ○ Very dissatisfied

The following questions are about your house. We would like to know how satisfied you are with your home and whether you are experiencing any problems.

14. In general, how satisfied or dissatisfied are you with the general condition of your home?

- ◯ Very satisfied
- ◯ Fairly satisfied
- ◯ Neither satisfied nor dissatisfied
- ◯ Fairly dissatisfied
- ◯ Very dissatisfied

15.Overall, how satisfied or dissatisfied are you with your home as a place to live?

- ◯ Very satisfied
- ◯ Fairly satisfied
- ◯ Neither satisfied nor dissatisfied
- ◯ Fairly dissatisfied
- ◯ Very dissatisfied

16.Are you currently experience any of the following within your home? (Tick all that apply)

- ☐ Condensation
- ☐ Lack of adequate heating
- ☐ Draught
- ☐ Damp walls and/or floors
- ☐ Mould
- ☐ Noise from neighbours
- ☐ Dry air
- ☐ Damp air
- ☐ Stuffy air
- ☐ Unpleasant odour
- ☐ Dust
- ☐ Static electricity, causing shocks

Now, we would like to ask you a few questions about your neighbourhood.

17. Overall, how satisfied or dissatisfied are you with your neighbourhood as a place to live?

- ○ Very satisfied
- ○ Fairly satisfied
- ○ Neither satisfied nor dissatisfied
- ○ Fairly dissatisfied
- ○ Very dissatisfied

18. To what extent do you agree or disagree with the following statements?

	Strongly agree	Somewhat agree	Neither agree nor disagree	Somewhat disagree	Strongly disagree
Overall, I am attached to living in this neighbourhood	○	○	○	○	○
I feel like I belong in this neighbourhood	○	○	○	○	○
The friendships and associations I have in this neighbourhood mean a lot to me	○	○	○	○	○

The next questions are about whether you recently have made any changes to improve the quality of the air in your home.

19. In the past month, have you made any changes to improve the air quality in your house?

- ◯ Yes, I have
- ◯ No, but I plan to
- ◯ No, and I don't plan to

Display This Question:

If The next questions are about whether you recently have made any changes to improve the quality of... = Yes, I have

20. What changes have you made to improve the air quality in your home?

- ☐ Cooking behaviour ____
- ☐ Heating behaviour ____
- ☐ Cleaning behaviour ____
- ☐ Personal care behaviour ____
- ☐ Smoking behaviour ____
- ☐ Other (fragrances) ____
- ☐ Other (window opening) ____
- ☐ Air purifier ____

Finally, we have a couple of questions about your health. Please note that any information you provide will be kept confidential. And you do not need to answer the questions if you do not want to.

21. In general, would you say your health is

- ◯ Excellent
- ◯ Very good
- ◯ Good
- ◯ Fair
- ◯ Poor

22.In the past month/four weeks have you had any of the following? [Tick all that apply]

- ☐ Coughing
- ☐ Bringing up phlegm
- ☐ Shortness of breath
- ☐ Wheezing attack
- ☐ Chest tightness
- ☐ Runny nose
- ☐ Blocked nose
- ☐ Sinus swelling
- ☐ Sneezing
- ☐ Sore throat
